# Dose rationale for gabapentin and tramadol in pediatric patients with chronic pain

**DOI:** 10.1002/prp2.1138

**Published:** 2023-10-06

**Authors:** Paul Healy, Luka Verrest, Mariagrazia Felisi, Adriana Ceci, Oscar Della Pasqua, GAPP Consortium, Corinne Alberti, Donato Bonifazi, Adriana Ceci, Thomas G. de Leeuw, Saskia N. de Wildt, Mariagrazia Felisi, Florentia Kaguelidou, Enora Le Roux, Rebecca Lundin, Laura Mangiarini, Dick Tibboel

**Affiliations:** ^1^ Clinical Pharmacology & Therapeutics Group, School of Pharmacy University College London London UK; ^2^ CVBF–Consorzio per le Valutazioni Biologiche e Farmacologiche Pavia Italy; ^3^ Fondazione per la Ricerca Farmacologica Gianni Benzi onlus Valenzano Italy

**Keywords:** dose rationale, extrapolation, gabapentin, pediatric chronic pain, pharmacokinetics, tramadol

## Abstract

Despite off‐label use, the efficacy and safety of gabapentin and tramadol in pediatric patients (3 months to <18 years old) diagnosed with chronic pain has not been characterized. However, generating evidence based on randomized clinical trials in this population has been extremely challenging. The current investigation illustrates the use of clinical trial simulations (CTSs) as a tool for optimizing doses and protocol design for a prospective investigation in pediatric patients with chronic pain. Pharmacokinetic (PK) modeling and CTSs were used to describe the PKs of gabapentin and tramadol in the target population. In the absence of biomarkers of analgesia, systemic exposure (AUC, Css) was used to guide dose selection under the assumption of a comparable exposure‐response (PKPD) relationship for either compound between adults and children. Two weight bands were identified for gabapentin, with doses titrated from 5 to 63 mg/kg. This yields gabapentin exposures (AUC_0–8_) of approximately 35 mg/L*h (1200 mg/day adult dose equivalent). For tramadol, median steady state concentrations between 200 and 300 ng/mL were achieved after doses of 2–5 mg/kg, but concentrations showed high interindividual variability. Simulation scenarios showed that titration steps are required to explore therapeutically relevant dose ranges taking into account the safety profile of both drugs. Gabapentin can be used t.i.d. at doses between 7–63 and 5–45 mg/kg for patients receiving gabapentin weighing <15 and ≥15 kg, respectively, whereas a t.i.d. regimen with doses between 1 and 5 mg/kg can be used for tramadol in patients who are not fast metabolisers.

## Graphical abstract

Exposure‐guided dose rationale for gabapentin and tramadol in pediatric chronic pain. Upper panels: Simulated concentration versus time profiles of gabapentin (A) and tramadol (B) on the last day of each titration step. Data are shown stratified by weight band for gabapentin. Solid line shows the median, shaded area indicates the 95% confidence interval. Lower panels: Whisker‐box plots showing the predicted AUC for gabapentin (C) and predicted AUC for tramadol (D) for each titration step. Gabapentin results are compared to the observed AUC values after doses of 1.2–3.6 g to adult subjects. Dotted red line shows the putative target exposure range for gabapentin (32.8–75.1 mg/L*h) and tramadol (1600–2400 ng/mL*h). Lower and upper hinges of the boxcplots correspond to the first and third quartiles. The upper whisker extends from the hinge to the highest value that is within 1.5*IQR of the hinge, where IQR is the inter‐quartile range, or distance between the first and third quartiles. The lower whisker extends from the hinge to the lowest value within 1.5*IQR of the hinge. Data beyond the end of the whiskers are outliers and plotted as points. IQR, interquartile range.

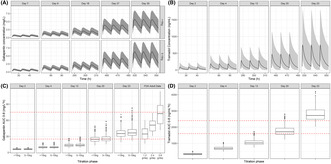

**Abbreviations**
AUC: area under the concentration vs. time curve
BMI: body mass index
BSA: body surface area
CLe: clearance
CLf: apparent formation clearance
Cmax: maximum concentration
CrCL: creatinine clearance
Css: concentration at steady state
CTSs: clinical trial simulations
F: bioavailability
FLACC: face, legs, activity, cry, consolability behavioral pain scale
FPS‐R: faces pain scale ‐ revised
Ka: absorption rate constant
K12, K21: intercompartmental transfer rate constants
NRS‐11: numeric pain rating scale
PD: pharmacodynamics
PK: pharmacokinetic
PKs: pharmacokinetics
PKPD: pharmacokinetic‐pharmacodynamic
q.i.d.: quater in die, four times a day
t.i.d.: ter in die, three times a day
V: volume of distribution

## 1 INTRODUCTION

Chronic pain, defined as pain lasting longer than 3 months as a continuous or recurrent condition affects the pediatric population. Yet, data supporting the clinical management of symptoms in this population is limited, as compared to adults.^1^ In spite of a treatment algorithm for neuropathic pain in adult patients, based on the severity of symptoms and treatment response, the same drugs and dosing regimens are used off‐label in children^2^ without formal evaluation or pharmacological rationale. This practice exposes pediatric patients to potentially non‐efficacious treatments and unnecessary risks, such as dosing errors and adverse drug reactions (ADRs). In fact, such a concern has been highlighted by a dedicated working group, where the most frequently used drugs in pediatric palliative care are described.^3^

The GAPP consortium was created to address some of these issues via the implementation of randomized clinical studies and retrospective and prospective pharmacovigilance data collection on the efficacy and safety of gabapentin. Gabapentin's analgesic effect has been found to be associated with voltage‐gated Ca^2+^ channel inhibition, which results in decreased presynaptic release of excitatory neurotransmitters. It has been approved for the treatment of neuropathic pain in adults at doses ranging between 900 and 3600 mg/day (t.i.d.). By contrast, its use off‐label to treat children with the same condition is based on a dosing regimen dose of up to 20 mg/kg three times daily for a child aged >2–12 years old (maximum single dose 600 g). For a child >12 years old, the maximum daily dose can be increased, according to response, up to a maximum of 3600 mg/day.^2^ To date, there is no data supporting the dose rationale in children. Similar to the evidence gap for gabapentin, there are no controlled pediatric clinical trial data on the efficacy of tramadol, a μ opioid receptor agonist, which also shows inhibitory effects on monoamine reuptake, various GPCRs, transporters, and ion channels. Despite the lack of marketing authorization for its use in children below 12 years of age in some countries, publications on the clinical experience with tramadol across a wide age group indicate that the recommended dosing regimen for children consists of a starting dose of 1–2 mg/kg/dose every 4–6 h with a maximum daily dosage of 8 mg/kg or 400 mg, whichever is lower.^4^, ^5^

Here, we focus on the scientific rationale and protocol requirements for the implementation of the GABA‐1 study (GABA‐1; NCT02722603),^6^ a non‐inferiority, phase‐III study aimed at assessing the efficacy and safety of a novel gabapentin liquid formulation (75 mg/mL) in children from 3 months to <18 years old affected by chronic neuropathic or mixed pain. In this trial, the investigative medicinal product (IMP) comparator is tramadol oral drops (100 mg/mL). As both drugs are titrated to a maintenance phase dose in clinical practice, the GABA‐1 protocol was designed to include titration, maintenance, and tapering phases according to a three‐times daily regimen. In addition, in contrast to standard efficacy protocols in pain research where systemic exposure is not evaluated, the pharmacokinetics (PKs) of both moieties will be characterized using sparse sampling and optimized sampling times.

There are inherent difficulties in conducting a trial of this nature. First, the incidence and diagnosis of such a heterogeneous condition makes it challenging to identify and recruit patients, some of whom may have already been exposed and failed to respond to the IMP. In addition, considering the age group of the trial, there is a need to measure pain using verbal and non‐verbal scales (FLACC, FPS‐R, or NRS‐11 pain scales),^7^ thereby introducing variation in pain measurements, including elements that may reflect not only nociception but also motor, sensory, and behavioral aspects of pain perception, which make the integration of the data across all age groups rather complex. This is further compounded by the use of rescue medication (which may lead to dropouts or blur the actual response to the IMP) and lack of a placebo arm (which if included as an intervention, would make the trial ethically questionable).

Based on the aforementioned points, it becomes evident why empirical treatment and dose selection may not be appropriate, and recommendations arising from off‐label use of gabapentin or tramadol cannot be easily generalized. Ideally, a stronger scientific rationale for the use of gabapentin along with dosing recommendations for the treatment of chronic, neuropathic pain would require the assessment of biomarkers of the pharmacological effects and an understanding of the relationship between these biomarkers and the anti‐nociceptive response.^8^, ^9^ In the absence of such biomarkers, and taking into account the symptomatic nature of the treatments, we have assumed comparable exposure–response relationships as a working hypothesis, even though differences in symptom severity and scales of pain may differ across age groups. While this assumption may not be fully aligned with current regulatory guidelines for extrapolation and bridging of efficacy from adults to children, they promote the implementation of a protocol in which PK–pharmacodynamic (PKPD) principles underpin the dose rationale.^10^, ^11^, ^12^ Furthermore, this assumption impels us to carefully consider changes in PKs due to developmental growth, organ function, and maturation processes across the different age groups.

Therefore, the current investigation is aimed at identifying opportunities to optimize the experimental protocol design and establish the dose rationale for gabapentin and tramadol in children. We use PK modeling and extrapolation concepts in conjunction with clinical trial simulations (CTSs) to ensure the implementation of a highly informative protocol. Our approach relies on the assumption that, despite heterogeneity between populations, the PKPD relationships of gabapentin and tramadol are comparable between adults and children. Therefore, by determining the efficacious plasma levels of these drugs in adults we can, to a degree of certainty, derive appropriate dose levels, and titration steps for pediatric patients.

## 2 METHODS

### 2.1 Population PKs and target exposure

Initially, population PK models of gabapentin and tramadol in adults and children were retrieved from the published literature.^13^, ^14^ These models were subsequently used to derive systemic exposure estimates for dose levels associated with the overall efficacy and safety profile of the moieties in adults. If necessary, models were re‐parameterised to ensure subsequent extrapolation of PK properties from adults to children taking into account developmental growth and maturation processes. Secondary PK parameters were derived as metrics of interest for the purposes of this analysis and included the area under the concentration versus time curve (AUC), maximum concentration (Cmax) and steady‐state concentration (Css).

To establish a target exposure range for gabapentin, data from an NDA submission (Neurontin®) to the USA Food and Drug Administration were used, in which the exposure and dose–exposure–response relationship have been evaluated^15^ in 690 patients with post‐herpetic neuralgia (Figure S1). Based on the evidence from clinical trials in adult patients, the therapeutic dose range (which ranges from 1200 to 3600 mg/day) appears to correspond to a mean systemic exposure at steady state of >32.8 μg/mL*h; this threshold was selected as the target exposure for the pediatric population under the assumption that total exposure is a clinically relevant driver or determinant of the analgesic response in chronic pain. Similarly, to define a target exposure range for tramadol, data from the clinical literature in acute pain were used, which included the PKs of the parent drug and its metabolite in adults and pediatric patients.^14^ Mean exposure was assumed to be a clinically relevant driver of the anti‐nociceptive response in chronic, neuropathic pain conditions. However, to address safety concerns and the potential effect of peak concentrations, mean steady‐state concentration was used instead of AUC. Tramadol concentrations between 200 and 300 ng/mL (from Garrido et al.)^14^ were used as a target range for the pediatric population, corresponding to an AUC range of 1600–2400 ng/mL*h.

### 2.2 Extrapolation of the PKs of gabapentin and tramadol

The extrapolation of the disposition parameters of gabapentin and subsequent implementation of simulation scenarios describing the changes in exposure associated with the proposed titration and maintenance phase of the clinical study protocol were based on the pediatric PK model reported by Ouellet et al.^13^

The covariate model was adapted to allow allometric scaling of the PK parameters (Figure S2). Given the renal elimination mechanisms associated with the clearance of gabapentin, changes due to ontogeny or organ maturation were described by creatinine clearance. An overview of the final estimates used in the simulated scenarios is shown in Table S2.

The extrapolation of the PKs of tramadol was based on the population PK model published by Garrido et al., ^14^ in which the disposition of the parent drug and its main metabolite were characterized. These parameters were used in conjunction with estimates of the absorption rate constant from Payne et al.,^16^ whose study population was administered tramadol oral drops, that is, the chosen formulation for the GABA‐1 study.

The final model represents the a two‐compartment PK profile along with one additional compartment, which represents the metabolite formation (M1) (shown in Figure S3). PKs was parameterised in terms of clearance (CL_e_), apparent formation clearance of M1 (CL_f_), volume of distribution (V), and the transfer rate constants (K_12_, K_21_), absorption rate constant (Ka) and oral bioavailability (F). Body weight was identified as a covariate on clearance and volume of distribution. Model diagnostics was evaluated by comparing model‐predicted versus observed profiles; this step provided evidence of data reproducibility and acceptable predictive performance. An overview of the final estimates used in the simulated scenarios is shown in Table S3.

### 2.3 Assumptions for the extrapolation of PKs from adults to children

For the sake of clarity, we enumerate the key assumptions underpinning the doses and dosing regimens which were derived for the pediatric population following scaling and extrapolation of the disposition parameters:

1. The therapeutic levels of both drugs in adults are also efficacious in children, that is, the PKPD relationship for the analgesic or anti‐hyperalgesic effects is the same, irrespective of the age of the patient (3 months to <18 years). Moreover, we assume that patients do not develop tolerance to the pharmacological effect during the course of the study.
2. As gabapentin has been mostly studied in pediatric patients with epilepsy, we assume that the PK disposition is not altered by the disease (i.e., chronic, neuropathic pain), whose pathological mechanisms should not have significant impact on organ function (including maturation and developmental growth).
3. Given the low prevalence of ultra‐rapid metabolic phenotype, patients will not be screened for polymorphism of CYP2D6. Therefore, genotype is not included as a covariate during the evaluation of the PKs of tramadol.
4. In the absence of data suggesting otherwise, simulations of drug levels over the course of treatment will be performed assuming that there is no metabolic induction or inhibition; as such there is no inter‐occasion variability and clearance estimates will be considered to be constant over the course of treatment.
5. As PK and pharmacodynamic data for tramadol are available only in acute pain conditions, we assumed the same therapeutic window is also applicable and safe for chronic treatment/repeated dosing.
6. There is enough time between titration steps to assess the analgesic or anti‐hyperalgesic effects, even if response to increasing dose levels may be delayed. Absence of improvement is to be considered as lack of pharmacological effect due to inter‐individual variability.
7. Lastly, it is assumed that there is no carry‐over effect between titration steps. Consequently, estimates of a putative dose‐exposure‐response relationship can be derived without bias.

### 2.4 Clinical trial simulations

#### 2.4.1 Simulation scenarios

The PK models including parameter estimates obtained by the extrapolation procedures outlined above were used to simulate a range of different dosing scenarios, with 1000 simulations performed in each. The main objectives of these simulation scenarios were (1) to obtain insight into the underlying dose–exposure–response relationship during the titration phase and (2) to ensure that patients were exposed to appropriate drug levels during the maintenance phase of the study. Given that the total sample size of the study had been agreed with regulators, each simulated trial consisted of a virtual patient cohort of 94 subjects across the age range between 3 months and <18 years old. Full PK profiles (i.e., concentrations vs. time after dose) were simulated for each patient and the parameters of interest (AUC, Css, and Cmax) were subsequently derived. For gabapentin, a weight‐banded dosing regimen was selected based on physiological and practical considerations. For tramadol, the currently approved doses were administered, with a cap on the total daily dose in accordance with the summary of product characteristics of the product. However, to preserve the blinding and reduce the burden of treament, an assessment was performed of the implications of a t.i.d. regimen, as opposed to four times daily dosing (q.i.d.). Graphical and statistical summaries were used to compare the results across different dosing scenarios against the predefined target exposure range. No statistical hypothesis test was applied for the selection of recommended dosing regimens and titration steps to be used in the clinical study protocol. The choice was based primarily on the maximization of the proportion of patients achieving the target exposure range.

### 2.5 Assumptions underpinning the simulation scenarios and final recommendations

The following assumptions were made for selection of the titration steps, dosing regimens, and sampling schemes to be recommended for the final clinical study protocol:

1. Adherence to treatment was assumed to be high (>90%) and doses to be administered without significant deviations.
2. Demographic characteristics sampled from the patient pool were deemed to be representative of the general pediatric population with chronic, neuropathic pain.
3. Correlations between demographic characteristics and physiological processes associated with drug disposition were considered constant throughout the simulated treatment period.
4. Despite known differences in the safety profile of gabapentin and tramadol, we assumed that there were no patient dropouts. This also implies that the simulated scenarios do not consider cases in which frequent rescue medication is required, and which would have resulted in patient withdrawal from the study.
5. To ensure characterization of the PKs across all dose levels and derive accurate estimates of the parameters of interest, simulated scenarios were based on the assumption that all patients reach the maintenance dose at the final titration step for both drugs.
6. Final recommendations of the dosing regimens to be used in the actual clinical study were based on mean estimates.

### 2.6 Virtual patient population

A data set (*n* = 800) including age, sex, weight, and creatinine clearance (derived from serum creatine values, explained in Supporting Information) was created based on the population data available from NHANES^17^ and CALIPER^18^ databases (Figure S4). Demographic and clinical baseline characteristics were sampled from the patient pool (*N* = 94) and used for the implementation of CTS. These virtual patients were used not only for the CTS scenarios but also to assess model performance. To ensure the accuracy and precision of the estimates obtained by extrapolation of the parameters from the original population (i.e., children with epilepsy), the models were externally validated by digitizing published data and comparing observed and predicted concentrations using visual predictive checks (VPCs).^4^, ^19^

### 2.7 Dose rationale and protocol optimization

From the results obtained from the different simulation scenarios at varying doses of gabapentin and tramadol, it was possible to assess the impact of interindividual variability in drug levels and compare the predicted systemic exposure in the pediatric population with the data observed previously in adults and children. Based on the anticipated PK variability and knowledge from previous experience with titrating both gabapentin and tramadol in clinical practice, a five‐step titration scheme was proposed for the final protocol. It is worth mentioning that according to this protocol design not all patients are titrated to the maximum dose level. The use of titration steps until the desired response is observed allows one to account for inter‐individual differences in the PKs, pharmacodynamics and disease severity. This approach also ensures that the appropriate dose level is used during the maintenance phase of the study.

As PK sampling represents a critical step for the characterization of the dose–exposure–response relationship across the population, different sparse sampling schemes were evaluated to establish the impact of sampling windows on the precision of the estimates of clearance during the titration phase. Given parameter uncertainty, ED‐optimality principles, as implemented in PopED version 0.4.0 (Uppsala, Sweden),^20^, ^21^ were used initially to identify suitable sampling windows for a maximum of four samples per patient. Final recommendations were derived by simulation–re‐estimation procedures, during which comparisons were made between the impact of repeated sampling during a single visit versus spread across different visits. The two schemes were evaluated to confirm whether or not an indwelling catheter would be required. All modeling and simulation steps described here were performed using NONMEM v 7.4 (Icon Development Solutions, USA). Data formatting or manipulation, including preparation of graphical and statistical summaries were performed in R version 3.6.2 (R Development Group, Vienna).^22^

### 2.8 Optimized sampling windows

Due to the young age of the participants, repeated sampling cannot be implemented as in adults. There are ethical and practical challenges in drawing blood from children, which cannot be overlooked. Therefore, a sparse sampling routine is needed to spare patients from unnecessary discomfort and multiple venepunctures.

Initially, the identification of optimal experimental sampling windows was also performed using ED‐optimality concepts. ED‐optimality provided the opportunity to select the most informative sampling times, ensuring higher precision in parameter estimates and PK model identifiability, as compared to traditional protocol designs for adult populations, where frequent blood sampling can be used. In a subsequent step, a simulation‐re‐estimation procedure was implemented to assess the impact of variation in the sample collection window based on a scheme with four samples per patient: one pre‐dose and three post‐dose samples, at the following intervals: between 0 and 2, 2 and 4, and 4 and 6 h. Despite differences in the disposition of gabapentin and tramadol, the study was optimized primarily for gabapentin. Patients receiving tramadol would also be sampled within the same windows, even though these times might not be equally informative for the characterization of tramadol clearance. Further details on the methodology, including final model parameters are presented in the Supporting Information.

### 2.9 Nomenclature of targets and ligands

Key protein targets and ligands in this article are hyperlinked to corresponding entries in http://www.guidetopharmacology.org, the common portal for data from the IUPHAR/BPS Guide to PHARMACOLOGY,^23^ and are permanently archived in the Concise Guide to PHARMACOLOGY 2019/20.^24^

## 3 RESULTS

### 3.1 Extrapolation of the PKs in children

Parameter values reported in the publications^13^, ^14^ from which the PK models were constructed informed the model refinement and extrapolation steps, as well as the simulation scenarios described in the following sections. Details of the model parameterisation are summarized in the Supporting Information along with a description of the model performance for each drug. As shown in Figures S5, visual predictive checks indicate concordance between model‐predicted versus re‐estimated profiles based on sparse sampling; this step provided evidence of data reproducibility and acceptable predictive performance. Moreover, the use of t.i.d. regimen was found to be acceptable, with predicted maximum concentrations varying by approximately 20% or less (Figure S6).

### 3.2 Baseline characteristics of the pediatric population

To perform simulations, different weight bands, and cut‐off weights were considered, taking into account required volume intake and other practical aspects, such as the use of dosing syringes for smaller patients. Given the changes in renal function across the age range of interest, two weight‐bands were identified that enable a simple, easily implementable dosing regimen for gabapentin in children from 3 months to < 18 years old. Therefore, data are presented following stratification into two groups, namely, children between 5 and 15 kg (*n* = 400), and >15 kg (*n* = 400). An overview of the baseline clinical and demographic characteristics of both groups is shown in Table 1.

**TABLE 1:** Baseline demographic covariates used across the different simulation scenarios for the evaluation of the pharmacokinetics of gabapentin and tramadol in children from 3 months to <18 years of age.

	Children 5–15 kg (*n* = 400)	Children >15 kg (*n* = 400)	All children (*n* = 800)
Age (years)	2.0 (0.25–5)	8.0 (2–17)	4.0 (0.25–18)
Weight (kg)	12.7 (4.5–15)	25.7 (15.1–76.6)	15.1 (4.5–6.6)
Height (cm)	89.3 (58.8–106)	128.4 (92–181.3)	100.3 (58.8–181.3)
BMI (kg/m^2^)	15.8 (12.3–19)	16.2 (12.7–24.9)	16 (12.3–24.9)
BSA (m^2^)	0.55 (0.27–0.66)	0.98 (0.6–2)	0.64 (0.27–2)
CrCL (mL/min)	188.3 (79.4–281.5)	163.2 (92.4–276)	173.7 (79.4–281.5)

*Note*: Values shown are the medians along with the corresponding range.

### 3.3 Clinical trial simulations

**FIGURE 1:**
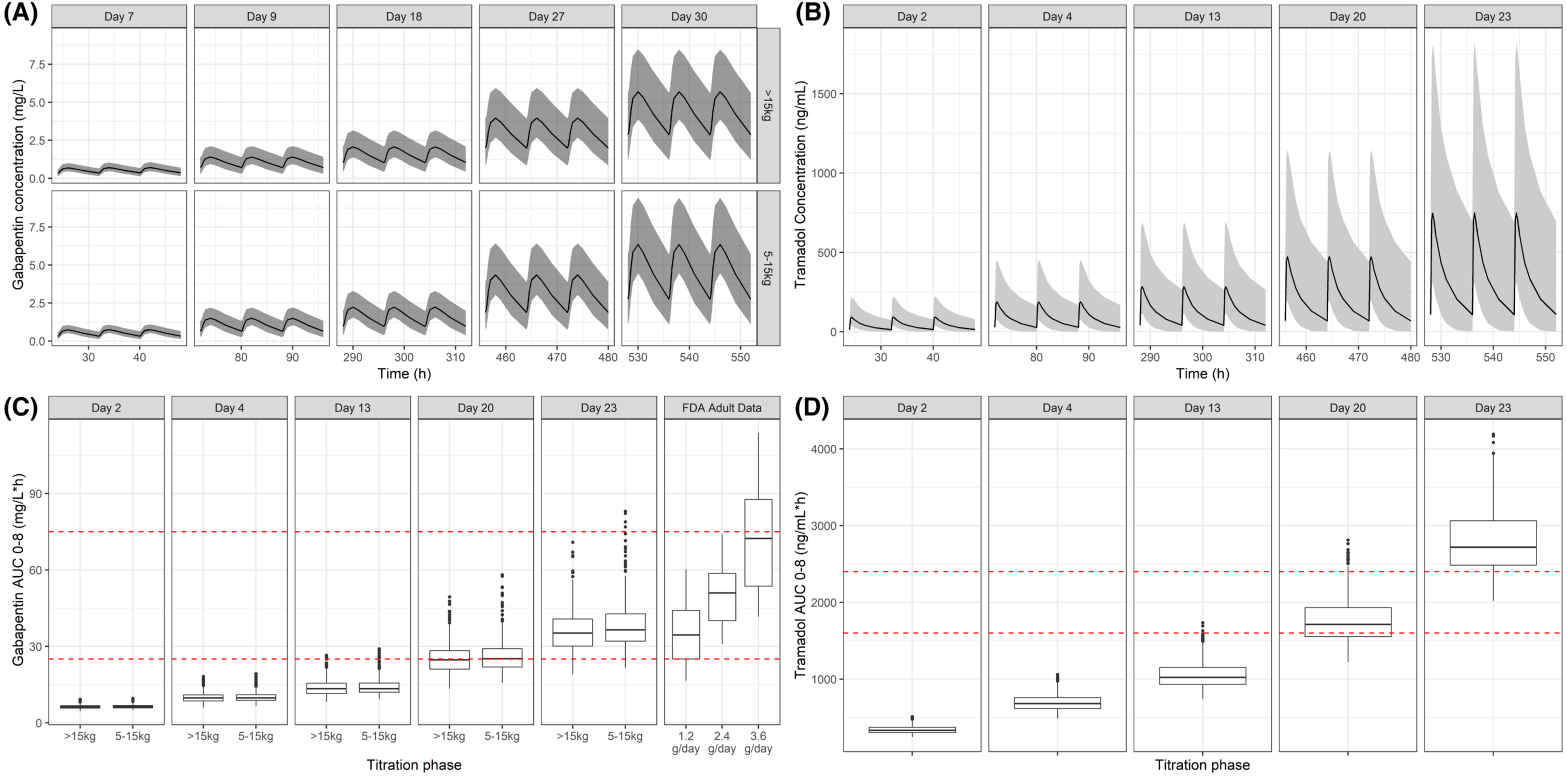
Upper panels: Simulated concentration versus time profiles of gabapentin (A) and tramadol (B) on the last day of each titration step. Data are shown stratified by weight band for gabapentin. Solid line shows the median, shaded area indicates the 95% confidence interval. Lower panels: Whisker‐box plots showing the predicted AUC for gabapentin (C) and predicted AUC for tramadol (D) for each titration step, based on a t.i.d. regimen. Gabapentin results are compared to the observed AUC values after doses of 1.2–3.6 g to adult subjects. Dotted red line shows the putative target exposure range for gabapentin (32.8–75.1 mg/L*h) and tramadol (1600–2400 ng/mL*h). Lower and upper hinges of the box‐plots correspond to the first and third quartiles. The upper whisker extends from the hinge to the highest value that is within 1.5*IQR of the hinge, where IQR is the inter‐quartile range, or distance between the first and third quartiles. The lower whisker extends from the hinge to the lowest value within 1.5*IQR of the hinge. Data beyond the end of the whiskers are outliers and plotted as points. IQR, interquartile range.

To mimic the proposed protocol (shown in Table 2), 94 patients were simulated per treatment arm. The results from the CTSs are shown in separate panels in Figure 1. In Figure 1A,B, every column has the last three dose intervals per titration step. The increase in exposure seen in the concentration versus time plots of both gabapentin and tramadol indicate that the chosen steps allow sufficient separation in exposure between dose levels for each. In Figure 1A, consistency in the concentration range achieved between the two weight groups can be seen throughout the titration steps for gabapentin. In Figure 1C,D, whisker‐boxplots show the exposures of both drugs for the proposed regimens. The regimen for gabapentin (on reaching the maintenance phase) corresponds to gabapentin adult exposures with an AUC_0–8_ of approximately 35 mg/L*h (1200 mg/day adult dose equivalent)^15^ for both weight groups. For tramadol, the results of the proposed regimen show that titration to response is necessary due to potential safety concerns, with mean C_SS_ reaching approximately 350 ng/mL for patients receiving 8 mg/kg, that is, mean values significantly above our postulated 200–300 ng/mL efficacy window. On the other hand, according to the most recent data of the International Association of Forensic Toxicologists, therapeutic blood levels of tramadol in adults range from 100 to 800 ng/mL, whereas the toxic level was defined to lie between 1000 and 2000 ng/mL.^25^, ^26^

**TABLE 2:** Recommended doses (mg/kg/day) during titration and maintenance phases. Recommendations are based on t.i.d. regimen for both drugs.

Investigational product	Weight group	Day 1	Day 3	Day 5	Day 14	Day 21
Gabapentin	5–15 kg	7	14	21	42	63
	>15 kg	5	10	15	30	45
Tramadol^a^	All patients	1	2	3	5	8

^a^ Maximum daily dose capped to 400 mg/day.

### 3.4 Dose recommendations for gabapentin

An outline of the final recommendation for the doses and dosing regimens to be used during the titration and maintenance phase of the study is shown in Table 2.

After repeated testing and fine‐tuning of the dose levels to be evaluated in each titration step, dosing regimens were identified which yield systemic exposure levels comparable to the target values in adults. These results are shown in Table 3. As can be seen across the different titration steps, there is a nonlinear increase in exposure with increasing doses of gabapentin.

**TABLE 3:** Overview of the final gabapentin dosing regimen administered as three daily dosing events, stratified by weight band.

Weight group	Study day	Dose (mg/kg)	Cmax (mg/L)	Css (mg/L)	AUC_0–8_ (mg/L*h)
>15 kg	2	5	2.8 (1.9–4.0)	0.8 (0.4–1.3)	6.3 (3.4–10.4)
5–15 kg	2	7	2.8 (2.0–4.0)	0.8 (0.4–1.3)	6.4 (3.5–10.4)
>15 kg	4	10	3.5 (2.4–5.0)	1.2 (0.7–2.2)	9.7 (5.2–17.6)
5–15 kg	4	14	3.5 (2.4–5.2)	1.2 (0.7–2.2)	9.9 (5.5–17.5)
>15 kg	13	15	4.1 (2.8–6.3)	1.7 (0.9–3.2)	13.4 (7.1–25.5)
5–15 kg	13	21	4.1 (2.9–6.4)	1.7 (0.9–3.2)	13.7 (7.6–25.5)
>15 kg	20	30	6.2 (4.0–10.1)	3.0 (1.6–5.9)	24.5 (12.9–47.0)
5–15 kg	20	42	6.6 (4.4–10.7)	3.2 (1.8–6.1)	25.6 (14.0–49.0)
>15 kg	23	45	8.3 (5.2–13.8)	4.4 (2.3–8.3)	35.0 (18.5–66.1)
5–15 kg	23	63	9.1 (5.8–15.3)	4.7 (2.6–8.9)	37.2 (20.4–71.2)

*Note*: Predicted exposure to gabapentin is summarized by secondary pharmacokinetic parameters at each dose level. Values shown are the medians and 95% confidence intervals.

### 3.5 Dose recommendations for tramadol

To ensure appropriate blinding and comparable titration steps in both study arms, different dose levels were considered over the same interval and number of titration steps for tramadol, as implemented for gabapentin. The dosing regimens which yield tramadol systemic exposure corresponding to the target values observed in acute pain are shown in Table 4. Due to safety considerations, genotyping may need to be implemented to assess polymorphisms in CYP2D6 and OCT1 transporter. As CYP2D6 fast metabolisers and loss of OCT1 activity are associated with clinically relevant increases in systemic exposure to tramadol and its metabolite, a lower starting dose of tramadol (0.5 mg/kg) should be considered when genotyping information is not available, followed by stepwise titration to the desired effect.

**TABLE 4:** Tramadol dosing regimen administered as three daily dosing events.

Study Day	Dose (mg/kg)	Cmax^a^ (ng/mL)	Css^a^ (ng/mL)	AUC_0–8_ (ng/mL*h)
Day 2	1	156.6 (74.7–331.5)	42.4 (13.9–110.0)	339.6 (111.2–880.0)
Day 4	2	319.1 (150.6–680.5)	86.5 (27.8–231.6)	691.9 (222.7–1852.6)
Day 13	3	482.7 (226.2–1041.2)	130.7 (41.7–363.4)	1046.2 (333.7–2907.4)
Day 20	5	803.7 (375.9–1735.0)	217.9 (69.3–604.8)	1743.1 (554.7–4838.7)
Day 23	8^b^	1275.5 (598.0–2740.7)	345.9 (110.9–948.7)	2767.2 (887.6–7590.2)

*Note*: Predicted exposure to tramadol is summarized by secondary pharmacokinetic parameters at each dose level during the titration and maintenance phases of the study. Values shown are the medians and 95% confidence intervals.

^a^ According to the International Association of Forensic Toxicologists, the therapeutic blood levels of tramadol in adults range from 100 to 800 ng/mL, whereas the toxic level was defined to lie between 1000 and 2000 ng/mL.^26^

^b^ Maximum daily dose capped at 400 mg/day.

## 4 DISCUSSION

To date, chronic, neuropathic pain conditions in children have been managed in an empirical manner, based on evidence of drug efficacy in adults and anecdotal reports of off‐label use in the pediatric population.^27^, ^28^ Since 2007, a new regulation has been in force in the European Union to ensure that evidence of efficacy and safety is obtained prior to approval of medicinal products for children.^29^, ^30^ Despite such a regulation, major challenges exist for the implementation of controlled clinical trials in rare or infrequent diseases or conditions.^31^, ^32^

The steps taken for the development of the GABA‐1 study protocol highlight some of the key challenges sponsors and investigators have to face to ensure a strong scientific rationale for the dose, dosing regimen, and clinical management of the condition during the course of a study. In addition, our approach shows how quantitative clinical pharmacology principles and tools can be used to overcome some of these challenges, enabling the implementation of a robust, informative clinical trial protocol.

First, it is important to realize that even in cases where evidence for efficacy and safety is required, extrapolation concepts are needed to identify a clinically relevant dose range.^33^, ^34^ In the case of chronic, neuropathic pain, where the use of placebo control is ethically disputable, biomarkers of the pharmacological effect are not available, and clinical scales are age‐specific (and not validated), it becomes evident why the assessment of exposure‐response relationships can be critical. Insight into the underlying PKPD relationships overcomes to some extent the absence of a dedicated dose‐finding study in children, which in the case of chronic pain is ethically unacceptable.

Irrespective of the potential differences in disease and measurement instruments, pediatric investigators and clinical researchers need to realize that exposure is affected by age‐related changes in PKs, and as such dose and dosing regimens need to take them into account. Inferences about the potential response (i.e., symptomatic improvement) can be made based on assumptions and scenarios, which in turn can be evaluated and tested in silico using computer simulations prior to exposing patients to an intervention.

Three aspects are worth mentioning with regard to the use of a model‐based approach for the development of a pediatric protocol. The first one regards the value of available data from adults and other indications when evaluating the efficacy and safety of medicines in children. In fact, our group has previously shown the contribution of historical data for the analysis of pediatric data and optimization of study design when an increase in the number of subjects is not feasible.^35^ In this context, a recent meta‐analysis involving 124 drugs revealed different treatment effects for one drug and a difference in the magnitude of treatment effect in 13.^36^ The authors emphasize the role of randomized controlled trials but acknowledge numerous issues with pediatric study protocols, including small sample size, lack of clear details on dose adjustment and age, making it difficult to investigate the influence of these factors on the treatment benefit dissimilarities. As pointed out by Oostenbrink et al. in response to these findings, one of the reasons for the lack or differences in treatment benefit or the opposite, harm in a given pediatric group, but not the other, maybe simply inadequate dosing.^37^ Rarely, efficacy studies consider the evaluation of PKs as a proxy for efficacy and safety. PK and PKPD modeling and simulation provide a framework for evidence synthesis that can be used for planning and design of experiments. A second aspect refers to the parameterisation of physiological processes associated with developmental growth and organ maturation, which provide the appropriate basis for dose selection and adjustment, taking into account the relevant factors that determine changes in PKs and consequently alter systemic exposure.^11^, ^38^ Often, for practical reasons doses are defined as a fixed amount or delivered in mg/kg, without further assessment of the implications for systemic exposure across the different age groups. The third aspect is the possibility of exploring the effect of interindividual variability and heterogeneity on PKs, pharmacodynamics, efficacy, and safety through simulation scenarios, which allow for the inclusion of significantly larger groups or cohorts of patients than an actual trial.^39^ Such a scenario analysis provides insight into the effect of baseline (clinical, genetic, and demographic) characteristics on exposure and response. It also enables identification of optimal experimental conditions and offers an opportunity to mitigate risks, taking into account protocol deviations.^40^, ^41^

This investigation has also unraveled some important deficiencies regarding the pharmacological basis upon which gabapentin and tramadol have been approved for the treatment, respectively, of chronic and acute pain in adults. Currently, the summary of product characteristics of both drugs recommend the use of titration to response, including a maximum daily dose, but does not correlate efficacy and safety with systemic exposure. This adds complexity to any extrapolation attempt, as response has not been assessed in a strictly quantitative manner. Rather, efficacy is defined on the basis of observed differences between active treatment and placebo. Such a deficiency is more evident for tramadol, where pro‐arrhythmic effects are known to correlate with drug levels in plasma, but no details are available to provide insight into a putative therapeutic window.^42^, ^43^ By contrast, we have managed to identify an informative sampling schedule both for the assessment of PKs and response (i.e., pain scores), which will facilitate an exploratory evaluation of the underlying exposure–response relationship. Regardless of the use of different clinical scales across the different age groups, the data collected during titration and maintenance phases of the study can be linked to systemic exposure using a sparse sampling matrix including four samples per patient.

Although the primary intent of a non‐inferiority study is to demonstrate comparable efficacy and consequently exchangeability of the treatment arms, the assessment of potential differences in the safety and tolerability profile of the two interventions may not be feasible, depending on the frequency or incidence of the adverse events. Here, we have shown the advantages of a model‐based approach to integrate all relevant available data in support of the dose rationale. In addition, simulation scenarios offer an opportunity to evaluate and mitigate risks associated with dose or exposure‐dependent adverse events; it also allows the assessment of the anticipated benefit–risk balance in a prospective manner.^39^ This consideration is essential for safeguarding of patients, especially when severe and often serious events are of concern, such as opioid‐induced somnolence, apnoea, and respiratory depression.^44^ Furthermore, the evaluation of predicted concentration versus time profiles, and in particular of peak concentrations, allows careful assessment of the impact of different titration steps and stop criteria.

Clearly, our attempt to optimize the study protocol has some important limitations. First and foremost is the absence of suitable biomarkers, which could complement the subjective assessment of chronic pain conditions and allow the development of a mechanism‐based PKPD model. This is, of course, beyond our control and even if brain imaging techniques could be considered as markers of pharmacological effect, their use in young children is not feasible. Similarly, it is not possible to characterize CNS exposure to gabapentin or tramadol and consequently establish the relevance of plasma exposure as a valid proxy for target engagement. Another limitation is the inability of pain scoring, particularly in younger patients, to differentiate between analgesia and sedation, which may lead to inaccurate assessment of treatment response.^45^, ^46^

From a PK perspective, one may not ignore the fact that the study will use a new formulation of gabapentin, for which we have had to assume that bioavailability is comparable to the existing dosage forms. The same potential issue applies to tramadol, as tablets and oral drops may show slightly different bioavailability than what was used in the simulation scenarios. We also acknowledge that the contribution of genetic polymorphism (CYP2D6) to the clearance of tramadol has not been incorporated into the proposed scenarios. Yet, such a covariate can be easily evaluated by simulations, and thresholds identified for safe doses and metabolite levels. At last, we cannot overlook the implications of a study without a placebo arm. Any effort to establish correlations between exposure and pain relief (analgesic or anti‐hyperalgesic effect) will be compounded by the underlying placebo effect.

In summary, chronic pain (lasting three months or longer) can arise in the pediatric population in a variety of pathophysiological classifications. The evaluation of the efficacy and safety of gabapentin and tramadol in this population is fraught with practical, clinical, and ethical challenges. The current investigation has identified opportunities to optimize the experimental protocol design and establish the dose rationale for gabapentin and tramadol in children. Regardless of the limited information on the underlying exposure–response relationships, this approach shows how PK modeling, extrapolation, and CTSs can be used to develop a highly informative protocol, taking into account heterogeneity and variability in the population.

## AUTHOR CONTRIBUTIONS

Oscar Della Pasqua designed the research protocol, Paul Healy and Luka Verrest analyzed the data, Paul Healy and Oscar Della Pasqua wrote the manuscript, and Adriana Ceci, Mariagrazia Felisi, Paul Healy, and Oscar Della Pasqua contributed to the discussion and interpretation of the results.

## FUNDING INFORMATION

This investigation was supported by the Gabapentin in Pediatric Pain (GAPP) Consortium, which has received funding from the European Union's Seventh Framework Programme for research, technological development, and demonstration under grant agreement n° 602962.

## CONFLICT OF INTEREST STATEMENT

All authors declare no competing interests for this work.

## ETHICS STATEMENT

This article is based on an in silico modeling and simulation protocol. It does not contain any new studies with human participants or animals performed by any of the authors. All clinical data required for the creation of the virtual cohorts derived from publically available databases. As such, ethical approval was not required.

## Supporting information


Data S1

## Data Availability

Simulation data sets and model scripts are available on request from the authors.
