# Supplementary material for: Dose rationale for gabapentin and tramadol in pediatric patients with chronic pain
Source: Pharmacol Res Perspect. 2023 Oct 6;11(5):e01138. doi: 10.1002/prp2.1138 (PMC10558965; doi:10.1002/prp2.1138)
Supplement: Supplementary file 1 — Data S1 [file PRP2-11-e01138-s001.pdf]

Supplementary Material

Table of Contents

Methods 2

Gabapentin Target Exposure 2

Gabapentin Population Pharmacokinetic Model 3

Tramadol Target Exposure 5

Tramadol Population Pharmacokinetic Model 5

Demographic Characteristics - Virtual Population 6

Clinical Trial Simulations – Dose Rationale for Titration and Maintenance Phases 9

Tramadol Dosing Interval 10

References 11

Methods

Gabapentin Target Exposure

In the absence of biomarkers and evidence indicating that the mechanisms underpinning the anti-hyperalgesic effects of gabapentin would be age-dependent, doses used in the simulations and, ultimately in the proposed dosing regimen for pediatric patients, were based on the assumption that comparable exposure would result in similar pharmacological activity and potentially comparable clinical response. We have therefore referred to the available exposure-response curve in adult neuropathic pain patients, and identified the target exposure range that is associated with clinical efficacy.

Based on the Neurontin® NDA submission file [1] (**Figure S1**), it was possible to extract secondary PK parameters associated with each dose level in the dose ranging study performed in adult neuropathic pain patients. As shown in **Table S1**, exposure ranges associated with clinical efficacy in adults were used as target for pediatric patients.

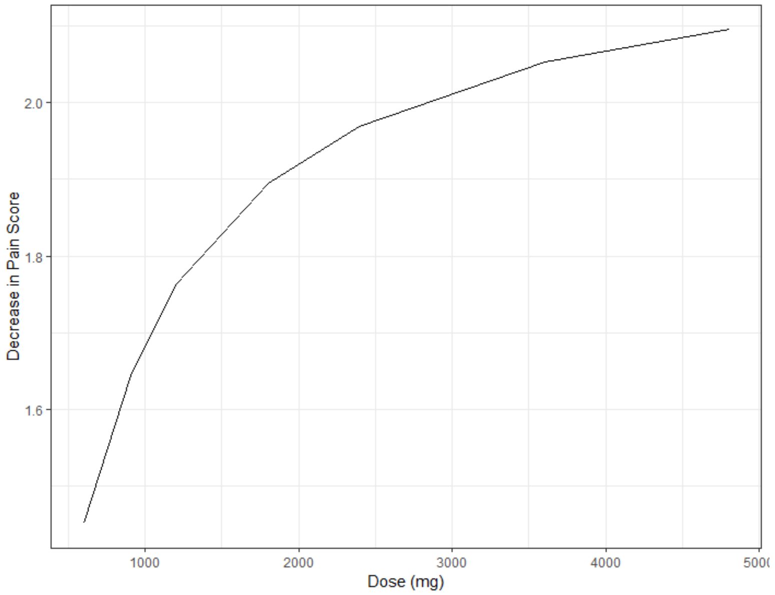

Figure S1. Gabapentin exposure-response relationship derived from dose ranging studies in adult neuropathic pain patients using the Likert pain scale (Neurontin®, NDA submission file[1]).

Table S1. Secondary PK parameters describing the systemic exposure to gabapentin after increasing doses of gabapentin. The data are presented as mean values. Cmax = maximum steady-state plasma concentration; AUC_{0-8} = area under the steady-state plasma concentration-time curve from 0 time until 8 h after administration; Ae% = percentage of dose eliminated as unchanged drug in urine.

Dose (mg/day)	Cmax (µg/mL)	AUC_{0-8} (µg.hr/mL)	Ae%
1200	5.43	32.8	47.2
2400	8.37	49.7	34.4
3600	11.6	96.5	32.5
4800	11.9	75.1	26.8

Gabapentin Population Pharmacokinetic Model

We selected the model reported by Ouellet *et al.* [2] for subsequent extrapolation of the pharmacokinetics of gabapentin in children and implementation of simulation scenarios in children with chronic neuropathic pain. The basic structural model, shown in **Figure S2**, is a one-compartment model with estimates of oral clearance (CL/F), central volume of distribution (Vd/F), first-order rate of absorption constant (ka), and lag-time prior to absorption (Tlag). Inter-subject variability of all PK parameters was described using an exponential model. Residual variability was described by combined additive and proportional errors. Covariates included in the model by Ouellet *et al.* were creatinine clearance and race on oral clearance, and body weight and population (epileptic patients or healthy subjects) on volume of distribution.

It should be noted that as the bioavailability of gabapentin is not dose proportional, changes in exposure due to variable F were described according to the following equation:

$F=(2340*Dose/(3080+Dose))/Dose$ *(equation 1)*

where F is the relative bioavailability of gabapentin. We have assumed that the dose-dependent decrease in bioavailability also applies to the paediatric population, irrespective of the use of a liquid dosage form.

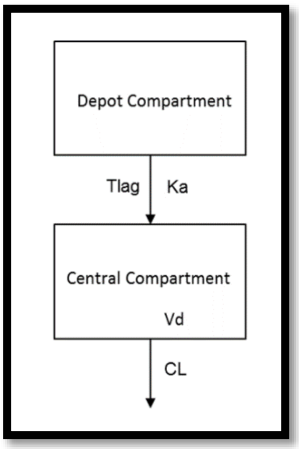

Figure S2. Diagram of the final population PK model describing the disposition of gabapentin. CL = clearance; Vd = central volume of distribution; Ka = first order absorption rate constant; Tlag = absorption lag time. Covariates include creatinine clearance on oral clearance (CL), and body weight on volume of distribution (Vd).

Table S2. Parameter estimates of the final pharmacokinetic model used in the simulation scenarios describing the systemic exposure to gabapentin during titration and maintenance phases of treatment in paediatric chronic pain patients.

Parameter (Units)	Notation	Population estimates (95% CI)
**CL=θ1*CRCL (L/h)**	θ1	0.116 (0.110–0.122)
**V=(θ2+θ3*WT)*2.05 (L)**	θ2	3.26 (−0.68–7.20)
	θ3	1.34 (1.13–1.55)
**Ka (min^{-1})**	θ4	1.24 (1.01–1.47)
**Tlag (h)**	θ5	0.181 (0.106–0.256)
**Inter-individual variability**		
**CL**	Ω1	0.088
**Vd**	Ω2	0.094
**Ka**	Ω3	0.185
**Tlag**	Ω4	0.626
**Residual variability**		
**Additive (µg/mL)**	σ1	1.32
**Proportional**	σ2	0.064

Abbreviations: Cl = clearance; Vd = central volume of distribution; Ka = first order absorption rate constant; Tlag = absorption lag time. Parameter estimates are summarized together with their 95% confidence intervals.

Tramadol Target Exposure

Tramadol is commonly prescribed for acute pain in paediatric patients, but it is not recommended for children below 12 years of age [3]. While the analgesic effects of tramadol at the currently approved doses has been shown to be dose dependent and associated with serum concentrations of 0.1-0.3 mg/L, there are no published data in chronic or neuropathic pain patients, in which pharmacokinetic data have been collected. Therefore, our working hypothesis is that nociception in chronic pain is determined by the same mechanisms underlying opioid activity in acute pain, irrespective of age. Differences in pharmacodynamic effects are more likely to occur due to phenomena such as tolerance.

Bearing in mind the large interindividual variability in the metabolism of tramadol, a dose rationale was proposed for paediatric patients with chronic pain, which allows up-titration to mean peak concentrations that correspond to the recommended titration regimen in adults, namely a maximum of 400 mg/day. This regimen ensures mean peak plasma concentrations of tramadol remain below 0.75 mg/L, a level which has not been associated with respiratory depression or other relevant adverse events.

Tramadol Population Pharmacokinetic Model

The population pharmacokinetic model published by Garrido *et al.* [4] was adapted for the purposes of our simulation work (**Table S3**). The disposition of tramadol in plasma was best characterized by a two-compartment model with one compartment for the metabolite (M1), shown in **Figure S3**. Keeping in mind the correlation between clearance and weight, the proposed adaptation improved model performance, reducing inter-individual variability and residual error when compared with the original model. The final model parameters included clearance (CLe), apparent formation clearance of M1 (CLf), volume of distribution (V), transfer rate constants (K12, K21), absorption rate constant (Ka) and bioavailability (F). Information on the absorption rate constant was incorporated into the model using the data from Payne *et*

al. [5], whose study population was administered tramadol oral drops (chosen formulation for the GABA-1 study protocol).

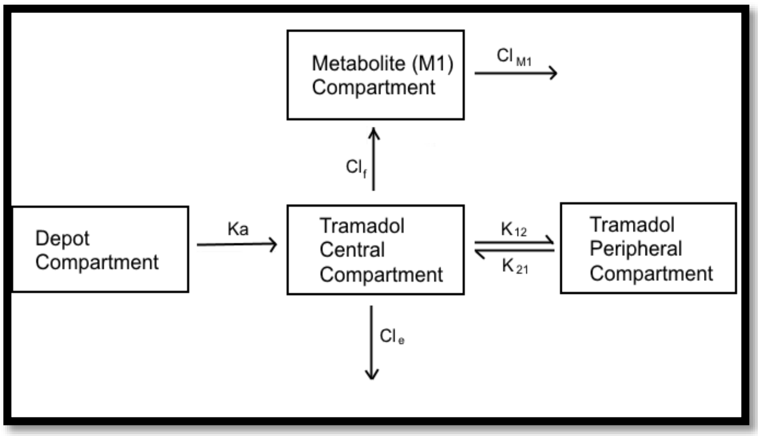

Figure S3. Diagram of the final population PK model describing the pharmacokinetics of tramadol. Ka = first order absorption rate constant; Clf = apparent formation clearance of the metabolite M1; Cle = clearance of tramadol; ClM1; clearance of the metabolite; K12 & K21 = transfer rate constants.

Demographic Characteristics - Virtual Population

A virtual cohort of patients was created for the purpose of this analysis using demographic and clinical baseline characteristics from three databases, namely, National Health and Nutrition Examination Survey of CDC (NHANES) [6], CALIPER [7] and the WHO [8]. Patient data between 3 months and 5 years were obtained from the WHO Child growth standards, whilst data for those aged between 2 years and 18 years old were retrieved from NHANES. Serum creatinine, which was required to calculate creatine clearance, was extracted from the CALIPER database.

Individual baseline characteristics were selected based on previously defined covariate factors known to affect the disposition of gabapentin and tramadol, namely: body weight, sex, height, age and serum creatinine. Creatinine clearance was required for the characterization of gabapentin disposition. It was calculated from serum creatinine values, stratified by age and sex; the Schwartz formula [9] was used for

patients aged 3 months to 12 years and the Cockcroft-Gault [10] formula for individuals aged 12 years and above.

Table S3. Parameter estimates of final pharmacokinetic model used in the simulation scenarios describing the systemic exposure to tramadol during titration and maintenance phases of treatment in paediatric chronic pain patients.

Parameter (Units)	Notation	Population Estimates (CI 95%)
**V = θ1+ θ2*(WT/19.64) (L)**	θ1	3.12 (0.4-7)
	θ2	4.89 (1.75-11)
**Clf (L/h)**	θ3	0.51 (0.21-1.2)
**Cle = θ4*WT/19.64) (L/h)**	θ4	14.7 (12.6-16.2)
**K_{12} (L/h)**	θ5	8.4 (5.1-12.6)
**K_{21} (L/h)**	θ6	1.76 (1.5-2.7)
**Ka (min^{-1})**	θ7	0.834
**F [x 100 (%)] (fraction)**	θ8]	0.68
**Interindividual variability**		
**V**	Ω1	0.1681
**Clf**	Ω2	0.1444
**Cle**	Ω3	0.0784
**K_{12}**	Ω4	0.2809
**Residual variability**		
**Additive error (ng/mL)**	σ1	43.01 (10-59)

Abbreviations: V = Central volume of distribution; WT = body weight; Clf = Apparent formation clearance of the metabolite, M1; Cle = Clearance of tramadol; K_{12} & K_{21} = transfer rate constants; Ka = First order absorption rate constant; F = Oral bioavailability; θ = PK parameter estimation; η = inter-individual variability; Ω = inter-individual or inter-occasion variability in population PK parameter; σ = population variance. Parameter estimates are listed together with their 95%-confidence intervals in parentheses.

The virtual patient cohort included a total of 1200 patients (**Figure S4**), with age varying from 3 months to 18 years old. Initially, the data set had three weight bands (5-15 kg, 15-30 kg and >30 kg with three equal groups of 400) to investigate any observed difference in gabapentin disposition between younger and older patients, even though such a stratification was not required for tramadol simulations. Two weight bands (5-15 kg and

>15 kg) were finally chosen as shown in **Figure 1** of the main text. Reference values of WHO and CDC were used to compare normal body weight, height and BMI values with those of our data set to ensure it reflected the known growth curves for male and female subjects. In addition, the data set was also checked for extreme values to prevent incongruous patient profiles being included, which would lead to skewed results. The workflow diagram in **Figure S4** displays the steps required for the creation of the virtual patient cohort.

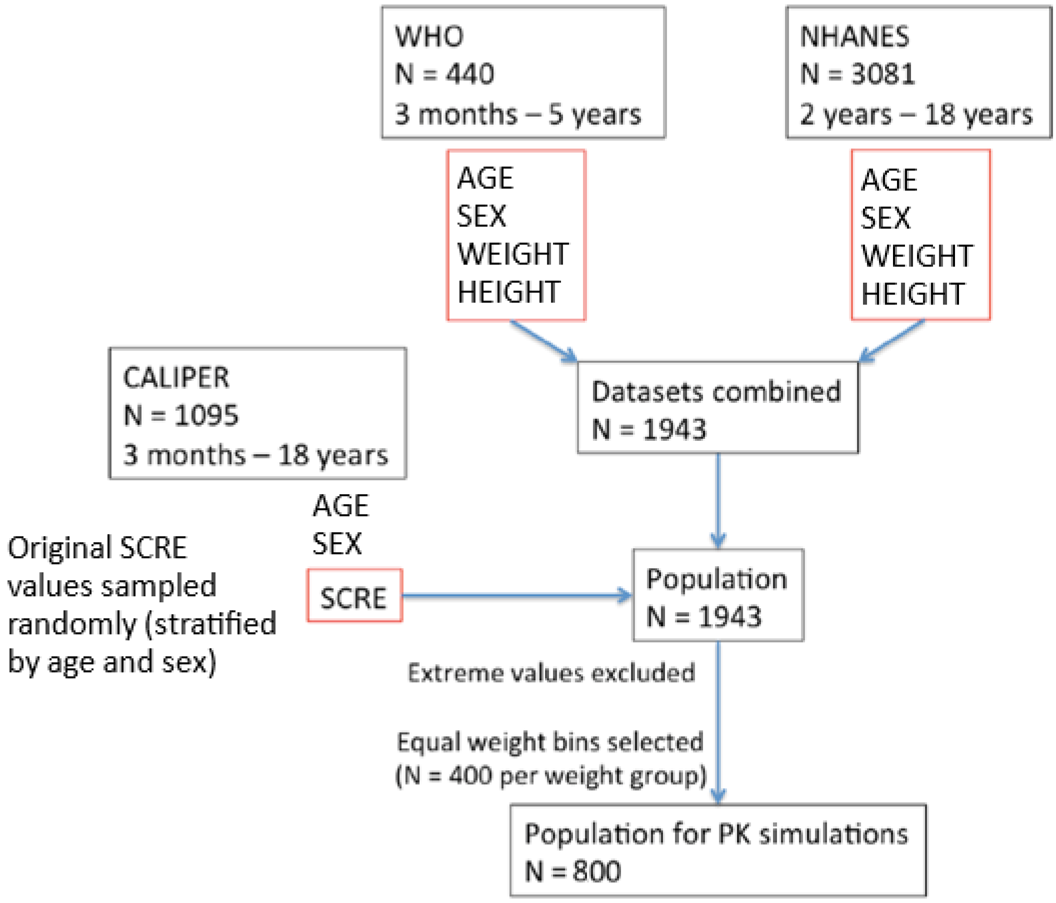

Figure S4. Steps required for the creation of a virtual patient cohort including predefined demographic and clinical baseline characteristics. SCRE; serum creatinine values.

Clinical Trial Simulations – Dose Rationale for Titration and Maintenance Phases

In addition to the identification and selection of suitable doses for up titration and maintenance phases of the protocol, attention was given to the optimization of blood sampling and assessment of gabapentin exposure in children. First model performance was assessed by comparing the anticipated profiles obtained with limited sampling (**Figure S5**). To ensure the proposed sampling scheme yields successful estimation of the parameters of interest, a simulation-estimation procedure was implemented using random sampling based on the recommended sampling intervals, with four samples per patient (n=94). Primary and secondary individual pharmacokinetic parameters were then derived using $PRIOR in NONMEM, with informative priors on all parameters.

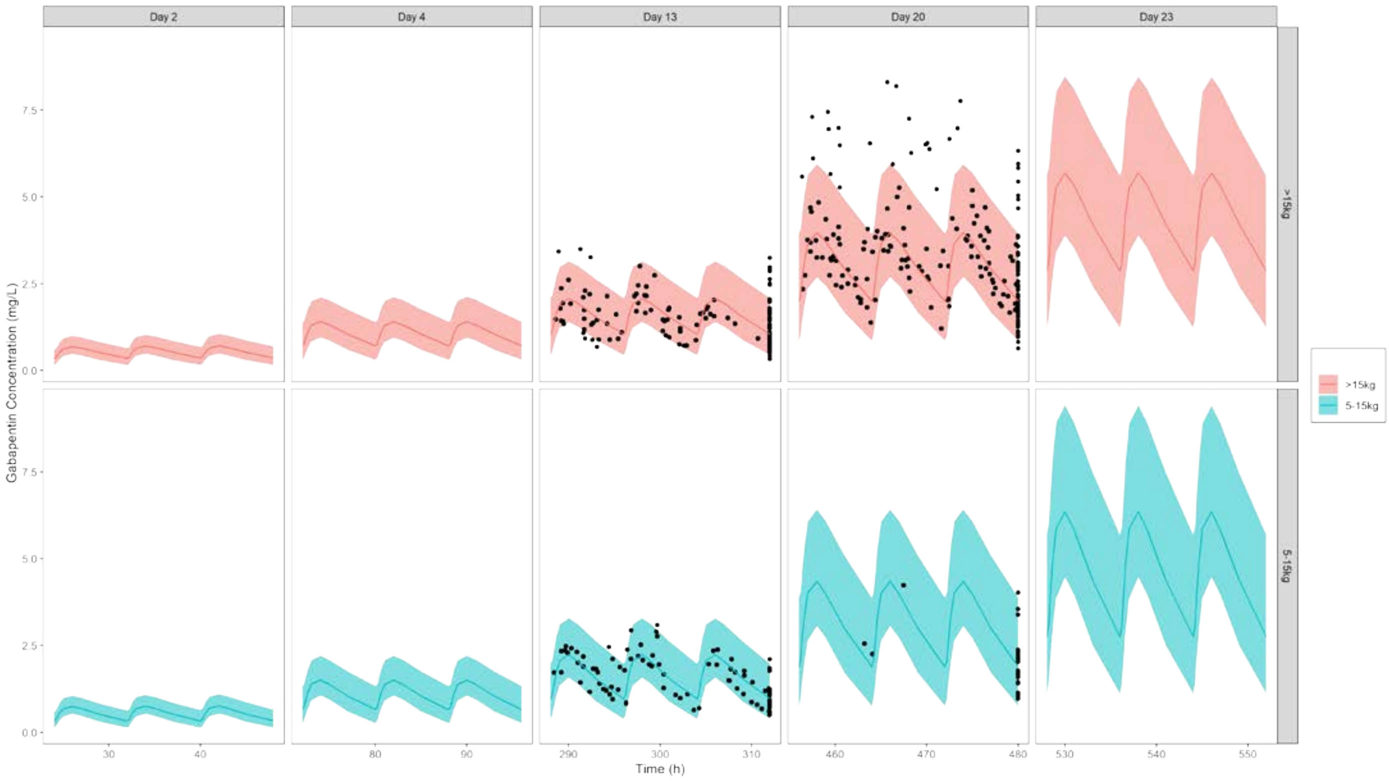

Figure S5. Simulated concentration vs. time profiles of gabapentin according to the proposed trial regimen. Panels depict profiles stratified by weight band. Solid line shows the median, shaded area indicates the 95% confidence interval. Black dots are random sampling points from simulated profiles (n=94) with re-estimated PK parameters using informative priors.

Tramadol Dosing Interval

To ensure the trial remains fully blinded, tramadol should be given three times a day to replicate gabapentin's dosing interval. Therefore, simulations were undertaken to assess the safety of shifting the dose from its recommended q.i.d. to a t.i.d. regimen using the maximal unit dose of tramadol for each scenario.

As shown in **Figure S6**, concentration vs. time profiles after an increase of 32% in tramadol amount per dose unit results in a proportional increase in peak concentrations and overall systemic exposure. This increase was deemed to be acceptable and clinically safe.

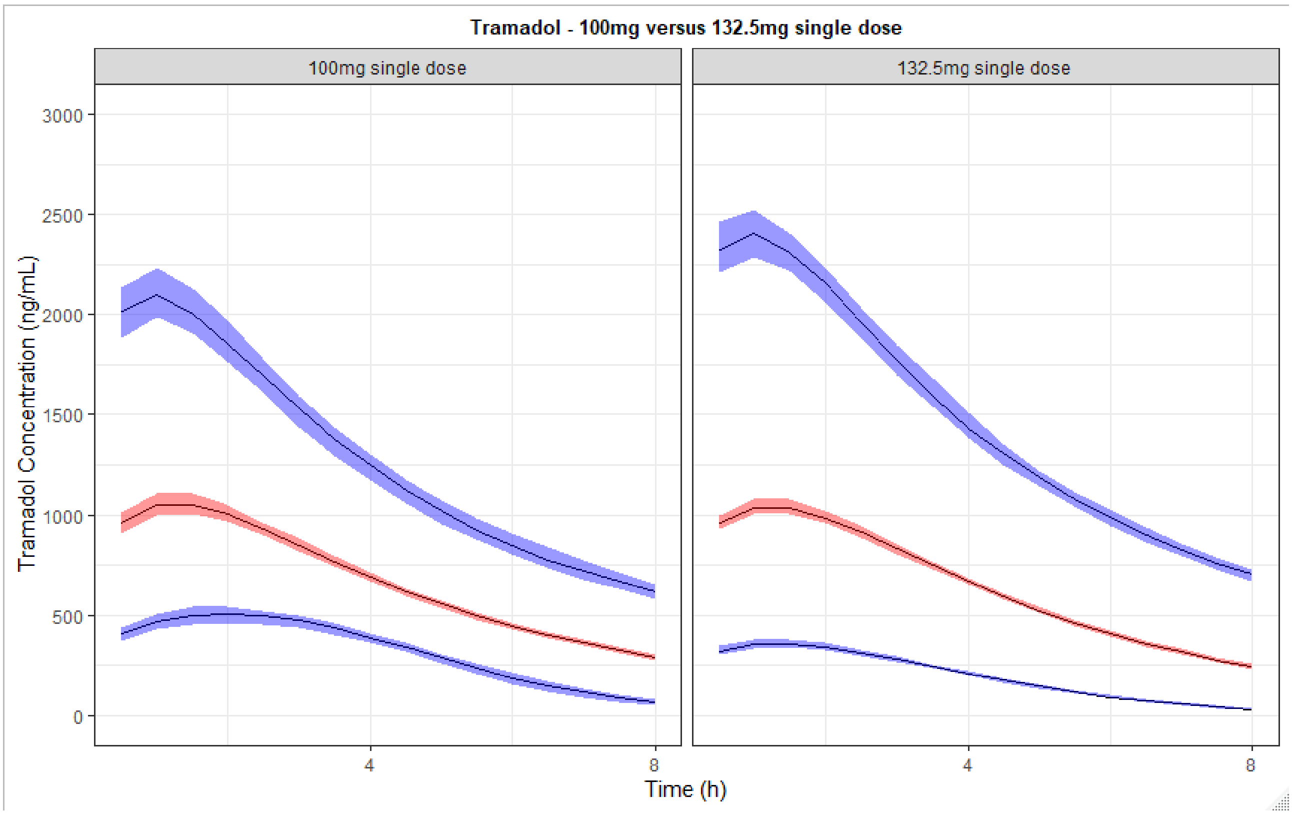

Figure S6. Plot comparing a single dose of tramadol at its recommended maximum single dose to the proposed maximal dose for t.i.d. dosing regimen. The t.i.d. regimen was required to ensure blinding of the treatment in the GABA-1 trial. Predicted concentration is shown as the median (soli red line) and 95% confidence interval (red shaded area) along with the 5th and 95th percentiles and the corresponding 95% confidence interval (black lines and blue shaded areas).

References

1. Neurontin NDA submission: 21-397. 07/08/2001. (www.accessdata.fda.gov/drugsatfda_docs/nda/2002/21-397.pdf_Neurontin_BioPharmr.pdf).

2. Ouellet D, Bockbrader H N, Wesche DL, Shapiro D Y, Garofalo E. Population pharmacokinetics of gabapentin in infants and children. Epilepsy Res. 2001; 47:229–41.

3. Joint Formulary Committee. British national formulary 78. (BMJ Publishing and the Royal Pharmaceutical Society, London, 2019-20).

4. Garrido MJ, Habre W, Rombout F, Trocóniz IF. Population pharmacokinetic/pharmacodynamic modelling of the analgesic effects of tramadol in pediatrics. Pharmaceutical Research 2006; 23(9): 2014–2023.

5. Payne KA, Roelofse JA, Shipton EA. Pharmacokinetics of oral tramadol drops for postoperative pain relief in children aged 4 to 7 years - a pilot study. Anesth Prog. 2002; 49(4): 109–112.

6. Centers for Disease Control and Prevention. Clinical Growth Charts. 2009; Available from: http://www.cdc.gov/growthcharts/clinical_charts.htm.

7. http://www.sickkids.ca/Caliperproject/Laboratory-professionals/index.html

8. WHO Child Growth Standards [Internet]. 2015. Available from: http://www.who.int/childgrowth/standards/en/.

9. Schwartz GJ, Muñoz A, Schneider MF, Mak RH, Kaskel F, Warady BA, Furth SL. New equations to estimate GFR in children with CKD. J Am Soc Nephrol. 2009; 20(3): 629-37.

10. Cockcroft DW, Gault MH. Prediction of creatinine clearance from serum creatinine. Nephron. 1976; 16(1):31-41.
